# Staying active after rehab: Physical activity perspectives with a spinal cord injury beyond functional gains

**DOI:** 10.1371/journal.pone.0265807

**Published:** 2022-03-23

**Authors:** Laura A. Baehr, Girija Kaimal, Shivayogi V. Hiremath, Zina Trost, Margaret Finley

**Affiliations:** 1 Department of Physical Therapy and Rehabilitation Science, Drexel University, Philadelphia, PA, United States of America; 2 Creative Arts Therapies Department, Drexel University, Philadelphia, PA, United States of America; 3 Department of Health and Rehabilitation Sciences, Temple University, Philadelphia, PA, United States of America; 4 Department of Physical Medicine and Rehabilitation, Virginia Commonwealth University, Richmond, VA, United States of America; Universitat Luzern, SWITZERLAND

## Abstract

Lifestyle physical activity following spinal cord injury (SCI) is critical for functional independence, mental wellness, and social participation, yet nearly 50% of individuals with SCI report no regular exercise. The objective of this study was to better understand factors leading to this participation gap by capturing the physical activity perspectives of individuals living with SCI. We completed small group interviews with nine individuals living with SCI across the United States. Iterative thematic analysis systematically revealed meaningful core concepts related to physical activity engagement with SCI. Emergent themes revealed challenges to lifestyle physical activity behavior including gaps in physical activity education, isolation during psychological adjustment, and knowledge limitations in community exercise settings. A secondary theme related to the COVID-19 pandemic emerged, highlighting additional environmental constraints affecting participation. Our findings suggest that most physical activity education is delivered during inpatient rehabilitation and is related to physical function. Lifetime physical activity strategies are achieved through self-education and peer networking. Personal motivators for physical activity include secondary condition prevention, while social and emotional barriers prevent regular adherence. These findings can inform the development and delivery of physical activity programs to maximize physical activity engagement in individuals living with chronic SCI.

## Introduction

Spinal cord injury (SCI) is often a physically traumatic event that has lifelong physiological, psychological, and social implications. Nearly 300,000 Americans are living with an SCI, often caused by accidents involving vehicles (38.2%), falls (32.3%), violence (14.3%), sports (7.8%), and medical/surgical complications (4.1%) [1]. While the cause of injury is often traumatic, partial to complete paralysis presents lifelong consequences. Individuals with SCI are predisposed to secondary health conditions that interchangeably impact the neurological, musculoskeletal, and cardiovascular systems, mental health (including anxiety and depression), as well as contribute to chronic pain, pressure wounds, urinary tracts infections, respiratory disorders and coronary disease [2]. The collective impact of these conditions leads to loss of function and independence, significant participation restrictions, and decreased quality of life [3]. Further, advancements in postinjury medical management have contributed to an increased average life expectancy in individuals with SCI approaching that of the general population, making the management of these conditions crucial to health and wellbeing over the lifetime.

Physical activity is imperative to reduce the risk of secondary health conditions and manage existing comorbidities [4]. Physiologically, individuals with SCI demonstrate decreased energy expenditure and metabolic response to exercise [5, 6], making physical activity participation more critical for overall health maintenance in this population. However, over half of individuals living with SCI report no regular physical activity [7]. Individuals with SCI are nearly 1.5 times more likely to lead sedentary lives than the general population [8] and demonstrate significantly lower physical activity levels compared to individuals with other chronic diseases at one-year post rehabilitation [9]. Unfortunately, physical activity levels continue to deteriorate during community reintegration [10], when adoption of lifestyle physical activity is crucial to avoid preventable medical complications. This participation gap presents a significant health crisis for individuals living with SCI and can initiate and exacerbate secondary conditions [4, 11] while decreasing psychological well-being and quality of life [2, 12]. Examination of systematic, personal, and environmental factors related to sedentary lifestyle is necessary to maximize health and quality of life for individuals with SCI.

It has been suggested that this dissonance between the benefits of physical activity and poor participation begins immediately following injury [13]. While the physical and psychological benefits of physical activity are a cornerstone of inpatient rehabilitation following SCI, initiation and maintenance of these recommendations following discharge is difficult to achieve [14–16]. Unfortunately, length of inpatient rehabilitation stay for individuals with SCI has been reduced by three times (2021; 30 days, 1970s; 98 days) over the last several decades [1]. Reduced rehabilitation time can contribute to a number of negative post discharge outcomes. For example, a longitudinal analysis demonstrated that depression at 3-months discharge was the strongest negative predictor of resilience [17], a critical factor to positive adaptation following SCI [18]. Decreased functional gains and psychological preparedness following shortened rehabilitation stay contributes to the challenge of lifestyle physical activity behavior adoption [13]. An unfortunate consequence is high rate of re-hospitalization; a prospective study found that 36% of individuals with SCI were re-hospitalized within one year of inpatient rehabilitation discharge [19].

To bridge this gap, a *Transformative Exercise* conceptual framework has been suggested to improve lifestyle physical activity behavior adoption through continuous professional oversight from rehabilitation through increasingly independent exercise settings [13]. However, practical progression between environments such as outpatient physical therapy and community fitness is challenging. Physical therapists recognize and educate on the value of physical activity to their patients with SCI, but promotion of community programs and physical activity education is lacking in clinical settings [20]. Lack of communication across rehabilitation and public settings negatively impacts effects of community-based education interventions for individuals with newly acquired SCI [21]. Physical and financial barriers like inaccessible facilities, transportation complications, and cost of equipment further limit physical activity participation [22, 23]. These barriers highlight health and community inclusion disparities for individuals with disabilities such as SCI. True community inclusion provides people living with SCI equitable opportunities for healthy living [24] including physical activity access, availability, and education.

One important way to enhance physical activity equity is to ground rehabilitation research and physical activity program development in the lived experience perspective of individuals with SCI. Identification of top priorities from the stakeholder perspective is critical to enhance efficiency and acceptability of interventions designed to enhance physical activity participation. However, there is limited participant-centered evidence on physical activity barriers and facilitators from the perspective of individuals with SCI [25]. A recent synthesis of qualitative evidence on experiences of SCI survivors revealed only six articles [26]. Further, physical activity program recommendations and preferences from the perspective of individuals with SCI have not been examined. Given the unexplored nature of physical activity preferences among individuals with SCI, a lived experience perspective and participant centered approach to research is warranted [27] to maximize benefit to the stakeholder. Participatory investigation, such as through the use of qualitative research methodology, is both underutilized in physical therapy research and greatly needed given our patient-centered approach to intervention and lifestyle behaviors [27]. The Physical Therapy Journal (PTJ) Editorial Board describes this gap; in 2018 only 4% of published original studies in the journal used qualitative methodology despite its immense value in understanding complex human experiences like physical activity participation with an SCI [28].

The purpose of this study was to begin to bridge this gap through identification of physical activity beliefs and recommendations from the perspective of individuals living with SCI. This study design utilizes thematic analysis as a meaningful first step toward improving lifestyle physical activity participation for individuals with SCI. The findings of this study will inform clinicians and wellness providers on the needs of individuals with SCI related to physical activity interventions. Further, the themes identified are intended to enhance the development and delivery of physical activity programs to promote long-term health and wellness for individuals living with SCI.

## Methods

An exploratory, qualitative descriptive study design was used to capture physical activity beliefs and attitudes from the unique perspective of living with an SCI [29]. Naturalistic inquiry of individuals living with chronic SCI was conducted through small group interviews. This design engaged participants in discussion on physical activity with SCI, which prompted reflections and explanations of the societal and personal phenomena that shape their experiences. The lived experience of physical activity engagement with chronic SCI was thematically analyzed. The Drexel University Institutional Review Board reviewed our protocol and deemed the study exempt. Consent was obtained verbally prior to initiation of group interviews. All recruitment and data collection activities were conducted via secure electronic communication software (Zoom Video Communications, 2020).

See Table 1 for demographic details on the study sample. A purposive sample of nine community-dwelling adults with chronic SCI (≥ 12 months) agreed to participate in the study. This sample size was chosen as the number of participants afforded the appropriate number of small group interviews to allow for thematic saturation and maximal generalizability [30, 31]. Electronic communication was used to inform participants of all study details. This convenience sample was collected through existing local and national partnerships as well as the Drexel University Research Website.

**Table 1:** Demographic features of study participants.

ID	Interview group	Age (years)	Sex	Racial group	SCI duration (years)	Level	Severity
A	1	36	Male	White	16	Cervical	Incomplete
B	1	41	Male	White	9	Thoracic	Incomplete
C	2	49	Female	White	18	Thoracic	Complete
D	3	28	Female	Asian/White	4	Thoracic	Incomplete
E	3	69	Male	White	34	Thoracic	Incomplete
F	4	70	Male	White	33	Thoracic	Complete
G	5	40	Female	African American	26	Cervical	Incomplete
H	5	33	Male	White	9	Thoracic	Complete
I	5	40	Male	White	6	Cervical	Incomplete

Qualitative validity and rigor were embedded using the Consolidated criteria for reporting qualitative research (COREQ) to maximize study design and reporting of findings [32, 33]. Objectivity was achieved through study team individual and collective assumption and bias reflection via memoing and bracketing at repeated study timepoints. This study was completed remotely to maintain health safety of participants and researchers during the COVID-19 pandemic. Participants were provided a secure web-based communication platform link prior to their assigned interview session and were asked to log on at a scheduled time. All participants were given the option to attend a one-on-one interview if preferred.

Prior to initiation of data collection, a standardized list of open-ended questions on physical activity with SCI were generated by the study team based on a-priori research questions and objectives. These questions were derived from available literature on physical activity following SCI as well as clinical expertise of the researchers. All study members completed reflective bracketing to minimize personal biases in question development. These questions served as topics anchors to maintain ongoing group discussion and maximize participant responses. Table 2 details specific questions used in all interviews.

**Table 2:** Open ended interview questions.

• “What type of physical activity do you enjoy?” • “What motivates you to participate in physical activity?” • “What challenges have you encountered to physical activity participation?” • “What types of physical activity would you like to in a program?” • “What would make it easier to participate in physical activity? • “What goals would you have for joining a physical activity program?”

During each interview, one member of the study team with previous experience in interview facilitation served as conversation moderator. This team member is a licensed physical therapist pursing a PhD in Rehabilitation Sciences with significant interest in community-based exercise for individuals with SCI. She did not have any relationships to the participants prior to study commencement. A second investigator was present during all interviews to generate additional field notes. Participants were oriented to the online room and provided general information on study goals. Conversational guidelines were set forth at the outset of all interviews. Participants were instructed to share all thoughts and opinions and were encouraged to engage in direct discussion with one another.

Participants were guided by the moderator through discussion using the pre-determined open-ended list of questions about physical activity with SCI. Questions were designed to elicit elaboration rather than “yes” or “no” answers. This maximized time that participants spoke to the group and in response to others, while minimizing influence of the study moderator. Each interview lasted, on average, 45–50 minutes. Each session was recorded via Zoom web-based communication software and saved on an encrypted server. Audio recordings were automatically transcribed via Microsoft Office Transcription software (Microsoft Office Version 16.48, 2021).

Thematic analysis as described by Braun and Clark [34] was completed to contextualize physical activity attitudes and beliefs from the perspectives of individuals living with SCI. Thematic analysis includes reflexive steps to achieve this goal including familiarization and transcription, code development, theme search, theme review, theme naming, and completion of report [34]. Transcripts were manually cleaned and de-identified by one study member to ensure deep familiarization with the data. Subsequently, the transcripts were reviewed with the rest of the team in the analysis phase. The code book was developed by consensus based on content areas specified in the interview guide. For example, the code “education” related to narrative responses describing personal experience with physical activity education. A second list of codes was generated based on emergent information that arose during group conversation outside of the interview guide. De-identified transcripts were entered into Dedoose, a qualitative analysis software for collaborative organizational purposes (Dedoose Version 8.0.35, 2018, Los Angeles, CA: SocioCultural Research Consultants, LLC). To maximize trustworthiness, search and review of themes was completed collaboratively through repeated meetings to discuss analysis until consensus of primary themes was reached.

## Results

Nine individuals living with chronic spinal cord injury (mean age 46 ± 16 years; 6 males/3 females; range injury duration 4–34 years) were interviewed through three small group interviews (n = 7) and two one-on-one interviews (n = 2). One-on-one interviews took place when additionally assigned participants for that block missed their scheduled time.

Thematic saturation (when repeated concepts arise across multiple interviews) was achieved and aligns with this previously suggested sample size for maximum generalizability [30, 31]

**Fig 1:**
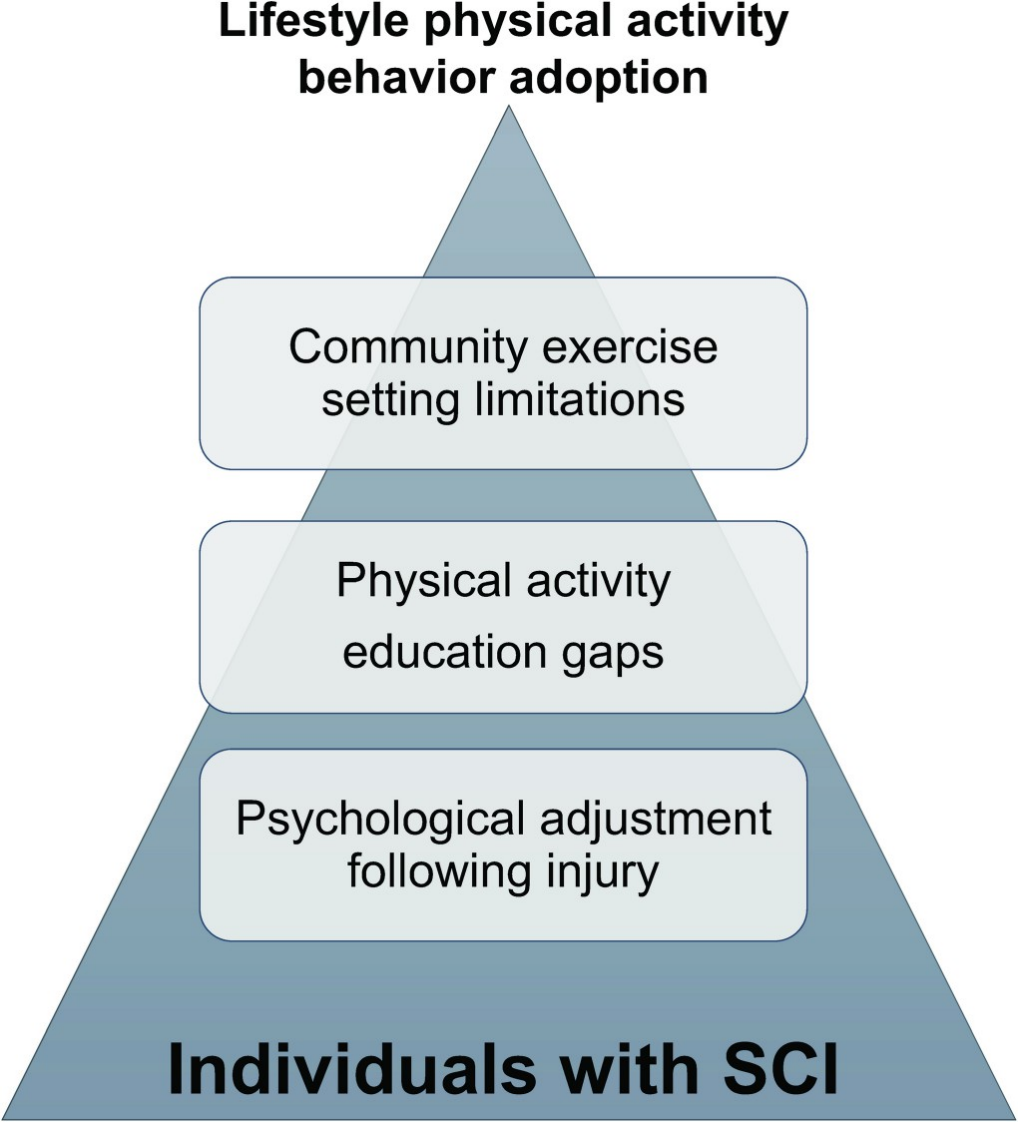
Challenges to physical activity lifestyle behavior adoption in individuals with SCI. Lifestyle physical activity adoption behavior is challenged by several personal and social factors including long-term psychological adjustment following spinal cord injury, gaps in physical activity education during community re-integration, and barriers in community exercise settings.

Findings indicated that individuals with SCI experience three types of challenges that make adoption of lifestyle physical activity behavior difficult. These include 1) psychological adjustment following injury, 2) gaps in physical activity education, and 3) community exercise setting limitations. Recommendations for community exercise opportunities were explored in response to these challenges. A secondary emergent theme arose relating to the impact of the COVID-19 pandemic on physical activity engagement. Fig 1. depicts these challenges in relation to one another. Table 1 lists participants by alphabetical pseudonym that denotes exemplar excerpts from interviews found below.

### Psychological adjustment following injury

An adjustment period following inpatient rehabilitation discharge was commonly discussed in relation to physical activity. Participants described a reprioritization of basic activities of daily living over enjoyable exercise as part of the physical and psychological trauma response.

*E*: *“It starts with how do I get this cup out of the closet and then you move from a cup to the plate*. *Then further down the line getting in a car and then further down the line*, *eating right and exercising*.*”*

*A*: *“You know*, *as a as a quadriplegic to transfer*, *to independently push around*, *you know*, *say*, *getting in a car*, *get out of car*, *wheelchair down like there’s a lot of things that it took a while to build my strength up to that level of physical activity that I wanted to partake in*, *especially like just pushing to and from campus*. *Those six blocks were rough at first*, *so you know the progression until I got to the point where*, *yeah*, *I I can do everything that I wanted to do*.*”*

Participants also acknowledged a collective understanding of a psychological transitional period from seeing themselves through a societal lens as disabled to empowerment as an individual with adapted physicality.

*D*: *“I also like kind of think about what other people are going to think about me doing stuff*. *So then I kind of get nervous and maybe they’re like judging me or like it’s hard for me to try new stuff because I don’t know if it’s gonna work and I don’t want to look stupid*.*”*

E: “*I don’t worry about what people think or look at me*. *I can’t control that I can only control me*, *but again*, *it took a long time to get to that point*.*”*

*E*: *“Being in a wheelchair is such a mental thing you know*, *to get your head around the wheelchair*, *not you know*, *to get your head around the idea that this is what I must do this is*, *this is how I have to conduct my life*. *This is how I need to go out and work everyday or I need to go out… It’s just it’s too easy to fall back and say*, *well I don’t feel good or I’m having a bad day or whatever*, *but I think if you can get your mind trained to fight*.*”*

### Physical activity education

Participants remembered the focus of inpatient rehabilitation being aimed at functional strategies that did not emphasize lifestyle physical activity.

A: “They (in rehabilitation) showed different types of strength building exercises, but not the ones that I’ve adapted as I’ve gotten further and further away from injury. Like the bare minimum things to increase likelihood of you getting independent, but once you hit independence, I feel like that kind of level of physical knowledge wasn’t really imparted.”

C: “I feel like it, like I said, it was mainly about how to maintain and navigate through your injury and um, just taking care of yourself, not necessarily exercise really wasn’t a part of it at all. We did exercise in rehab, but to me it wasn’t … It was never the main focus.”

E: “Now that I’m thinking back, I do feel like they told me like the importance of doing like shoulder exercises for like ’cause you know, you’re always pushing so it’s important to do, like do pull kind of work. But that definitely I heard from my OTs and PTs. But yeah, besides that I feel like I just do what I want to do and I guess I kind of tried to do like modify what I was doing before.”

Further, participants describe a steep physical activity informational decline following inpatient rehabilitation discharge. One participant described continuing to utilize information he received during inpatient rehabilitation a decade prior:

F: “I have articles that go back 10 years and I’ll still read them over.”

Other participants described self-education as their most prominent strategy to gain physical activity education. These often were peer and internet-based sources and many did not adequately address SCI-specific needs.

D: “The mental barriers that that we all feel it’s then you can go on the Internet and you can look up exercises and as XXXX says, you can do a YouTube thing and maybe you get 30% of that them that work for you. Even chair exercises. Maybe 30% of them work for you… Like if I go to YouTube an if I’m typing like you know, exercise in the chair, but then they would still do like marches in the chairs and then it’s like, oh that’s not gonna work either. So I guess just like finding stuff that I want to do is hard, so yeah usually, I just need to try to come up on my own.”

E: “I find it on the Internet, you know in in different things that have different sports magazines that I’ll read most of the stuff that does not apply to me.”

### Community exercise setting limitations

Despite rich discussions about the positive benefits of physical activity, participants repeatedly highlighted environmental and societal barriers that increase stigma and limit access to community physical activity engagement.

B: “…lack of accessibility on society’s part.”

D: “There are very few places that are adaptable for somebody that’s handicapped, especially for a wheelchair person.”

E: “They’re just not that many places for a person like myself, you know. And that’s unfortunate, but again. If more people were involved, more people from yes, yeah community were involved in this. We create a market. And that market doesn’t exist, so it becomes a self-fulfilling prophecy. Oh, there’s no place to go. Therefore I don’t workout. It will work out because there’s no place to go. So you know, and we oughta try to break that cycle somewhere.”

D: “It’s possible to adapt to people in wheelchair ’cause I don’t want to just show up and then the person leading the group be like, oh, I don’t know what to do with you and then it’s like awkward for everyone just like knowing that.”

E: “In any workout I like to, even when I’m wheeling for, you know through the streets or around the track, virtually nobody comes up to a guy in a wheelchair and says hey, why don’t you try that? It’s not that they don’t. Nobody comes up to you. You gotta seek them out.”

C: “I mean there is things that if the seat would have come off, I would have been able to get on it or get closer to it. I would try to explain to them look next time you order a new piece of equipment or something, maybe where the seat comes off. So somebody in a wheelchair can get in there or anybody with limited mobility, but they really didn’t want to hear it. They really just kind of brushed me off.”

B: “We’re normal people, but people don’t …the public doesn’t always view us like that. So I figure is more, the more I can be out and in everything the more normal I feel and I feel that people get used to seeing me and it kind of breaks the stigma of people in wheelchair.”

### Community-based exercise program recommendations

Recommendations for community exercise for SCI represented the highest frequency code application (9% of all codes). Geographic proximity, low financial burden, knowledgeable instruction, reliable scheduling, and socialization were top priorities for participants.

H: “If it’s gonna be far away that I’m gonna have to drive like you know 30–45 minutes and it’s like well, maybe I’ll just stay around and do stuff here. Yeah, and then if it’s gonna be virtual then it’s like am I gonna be able to do it at my apartment? You know space wise, equipment wise.”

D: “I don’t know like in my mind that’s what I miss the most like having like a group exercise session ’cause if it’s 1 on 1 then that’s nice too. But I can do it by myself, but I miss like those like group session where like we like push each other so that we are like work harder.”

I: “I would like to have you know somebody who really is knowledgeable. Just watch me from time to time and just say, OK, you know, try this or try that or something of that nature.”

Participants highlighted that the misconception that wheelchair users are deconditioned minimizes the effectiveness of readily available group exercise classes.

I: “But yeah, I’m pretty open to any kind of exercises and I’m happy to try new stuff, but for me, like high intensity is better than low intensity. So I guess if it’s gonna be like low key just stretching, then I’m probably not gonna join because I can stretch on my own.”

F: “Again, living in a retirement community, I’m actually one of the younger people here, so most of the people are more elderly and more limited in their physical active abilities, which is kind of strange for me to say, being in a wheelchair, but you know, I find that I can do more, at least with my upper body than a lot of people can here.”

### Impact of COVID-19

Participants also discussed consequences related to physical activity programming changes during the COVID-19 pandemic.

H: “Social interaction is huge, especially this COVID stuff is really … You know, as soon as I could get back to the gym. It was like, yeah, I’ll wear a mask. I don’t care like let me let me get out and see people, talk to people.”

G: “They work with me a lot especially with coronavirus. You get kinda. You can get kinda depressed ’cause you’re not able to go out. You know at this time like you know, sometimes you aren’t limited from doing things, but this could make it even worse so. And then because of your health situation, you had to be even more careful.”

H: “I average about two times a week (at the gym) until they shut down pre-COVID and they’re still not open, and I think that’s been a big challenge for me just because I’m stuck at home and there’s only so much you can do at home.”

## Discussion

The findings of this exploratory qualitative study indicate that individuals with SCI experience numerous challenges that impact lifestyle physical activity behavior adoption. Participants are aware of the physiological benefits of physical activity, but psychological and social factors outweigh this knowledge. Nearly 80% of individuals with SCI indicate that physical activity is important and express interest in maintaining an active lifestyle [23]. However, internal barriers such as motivation and negative perceptions of physical activity have a strong association with exercise participation [22, 23]. Physical and financial barriers including inaccessible facilities, transportation complications, and cost of equipment further limit physical activity participation [22, 23]. Despite these barriers, participants describe a personal journey with physical activity that Is often discovered through trial and error. This is in alignment with an SCI concept of finding self-value and confidence to create opportunities amidst restrictions [35].

Recommendations for community exercise strategies by participants are in alignment with currently available literature on the cascade of positive psychological effects related to socialization in exercise that fosters community. In a recent study, exercise participation created larger social networks [13] and increased social achievement [36]. Engagement in physical activity is positively associated with social quality of life [37, 38], reduction of depression and negative mood, increased self-confidence, improved body image, and enhanced quality of life [36, 39, 40]. Qualitative findings support these outcomes in individuals with SCI who reported that physical activity facilitated optimism and positive outlook, and helped manage stress, thus enhancing overall psychological well-being and mental health [22].

Participants desire exercise environments that facilitate psychosocial wellbeing, yet their perception is that physical activity education that they formally received in rehabilitation was aimed at function. This is critical as resilience is imperative to recovery following SCI, and is influenced by healthcare provider experiences supportive of self-efficacy [26]. These findings present an educational gap that rehabilitation and community exercise specialists can fill through embodiment to harness to facilitate lifestyle physical activity behavior. The concept of embodiment is centered on the understanding of the body as the ground for which subjective experiences takes place [41]. Therefore, the body is a vehicle for reflection and healing, as opposed to a functional vessel only. This is especially critical to individuals with SCI as most injuries are traumatic in nature. In rehabilitation research, the physiological trauma response of SCI related to inflammatory mediators is heavily studied [42–44], yet there is extremely limited evidence on interventions to target the *psychological* trauma response to injury, which our participants discussed at length in relation to physical activity. Physical embodiment modalities such as Yoga [45] and Tai Chi [46] foster physical and mental wellbeing in individuals with SCI, yet data to confirm these effects in large scale controlled trials are lacking. Adoption of these strategies early on in recovery as adjunct rehabilitation techniques, outpatient therapeutic tools, and condition-specific exercise programming is warranted to center embodiment as a valuable technique to foster psychological wellbeing that is critical to resilience and self-efficacy—personal factors imperative to long term physical activity adherence [47].

Further, psychologically-informed clinical and exercise intervention development and implementation should be prioritized for individuals with SCI. Psychologically-informed care addresses physical and psychosocial factors together through empirically supported interventions, such as cognitive-behavioral techniques [48]. Currently, psychologically-informed care research is primarily focused on orthopedic clinical populations such as low back pain [49]. However, this care technique should be examined for individuals with SCI due to their high rate of musculoskeletal pain [50], the inextricable relationship between the pain-response with the neurological system, and its value in addressing psychological trauma responses. Qualitative methodology is an important strategy to identify unique SCI-related psychosocial factors and provide a more complete picture of the needs of this population in psychologically-informed care development.

Physical activity interventions and education delivered by rehabilitation and exercise specialists is both needed and warranted beyond formal rehabilitation environments to foster lifestyle physical activity behavior. This reflects previously proposed strategies to increase physical activity participation by individuals with disabilities through continued professional oversight from rehabilitation to community facilities [13]. These interventions

should target the psychological and social benefits of physical activity in addition to functional implications. Geographic proximity, expert instruction, regular scheduling, and social engagement should be emphasized in program planning and implementation to bolster engagement and appropriateness for individuals with SCI.

Transferability of these results is limited as we interviewed nine participants. However, the demographic features of this sample are in alignment with national breakdown in the SCI population [1]. The repeatability of this study is limited as data was collected in the context of global pandemic, however, the physical activity-related findings support previous work [25].

## Conclusions

This study contextualizes the lived experience of SCI related to physical activity and program recommendations. These findings may explain and enhance quantitative findings from previously available research on physical activity with SCI. Further, these results can inform the development and delivery of physical activity programs to maximize physical activity engagement in individuals living with chronic SCI. Our findings suggest that most physical activity education is related to physical function and health maintenance and is delivered during inpatient rehabilitation by therapists. Lifetime physical activity strategies are achieved through self-education and peer networking. Motivators for physical activity engagement are aimed at independence maintenance and secondary condition prevention, while social and emotional barriers prevent regular adherence. Physical activity programs should minimize these barriers while bolstering social connectivity and lifelong physical activity education.

## Supporting information

S1 File Descriptive code (salient phrase based on language-based interview data) counts relating to major discussion points and emergent topics across each interview. (XLSX)
